# An ultrasound-assisted procedure for fast screening of mobile fractions of Cd, Pb and Ni in soil. Insight into method optimization and validation

**DOI:** 10.1007/s11356-016-7745-3

**Published:** 2016-09-27

**Authors:** Barbara Leśniewska, Marta Krymska, Ewelina Świerad, Józefa Wiater, Beata Godlewska-Żyłkiewicz

**Affiliations:** 1 Institute of Chemistry, University of Bialystok, K. Ciołkowskiego 1K, 15-245 Białystok, Poland; 2 Faculty of Civil and Environmental Engineering, Bialystok University of Technology, Wiejska 45E, 15-351 Białystok, Poland

**Keywords:** Ultrasound probe, Modified BCR sequential extraction, Environmental analysis, Uncertainty budget

## Abstract

A fast ultrasound-assisted sequential extraction (UASE) procedure for the determination of cadmium, lead and nickel fractions in soil was developed and fully validated. The working parameters of an ultrasound probe were optimized by comparing the content of metals in soil extracts obtained by the UASE procedure with that obtained by the conventional (with the aid of a vertical rotor) modified Community Bureau of Reference (BCR) procedure. The content of metals in soil fractions was determined by electrothermal atomic absorption spectrometry. The total time of extraction of metals from soil was shorten from 48 h to 27 min (total sonication time). The trueness of the developed method was confirmed by analysis of the certified reference material BCR-701. In order to indicate critical points of the developed UASE method, uncertainties of fractionation results were calculated and compared with those calculated for conventional modified BCR procedure. The method usefulness was tested for the determination of metal fractions in different types of soil collected in the Podlasie Province (Poland). The proposed procedure could be used for fast screening of mobile fractions of several heavy metals in soil.

## Introduction

The contamination of sediments, soils and plants by heavy metals is of major concern due to their toxicity and bioaccumulative nature. Many anthropogenic activities (e.g. combustion of oil and coal, chemical industry, ferrous and non-ferrous metal production, waste incineration) have resulted in the redistribution of cadmium, lead and nickel from the earth’s crust to the soil and other environmental compartments. Lead and cadmium are considered as toxic metals for plants and humans (Andresen and Küpper 2013; Pourrut et al. 2011). They can be absorbed from soil solution mainly through the roots and thereby may enter the food chain. Nickel, in low concentrations, fulfils a variety of essential roles in living organisms, e.g. as a constituent of several metal enzymes. However, excessive amount of nickel in soil and in nutrient solution is toxic to most plant species, affecting, e.g. nutrient absorption by roots, inhibiting photosynthesis and transpiration and causing ultrastructural modifications (Ahmad and Ashraf 2011). Total emission of these metals in 2013 in Poland was 15 t for Cd, 561 t for Pb and 148 t for Ni, while in the European Union was 63 t for Cd, 1836 t for Pb and 697 t for Ni (EEA Technical report no 8/ 2015). The excessive metal exposures result in reduced yields of agricultural crops.

Total metal content in polluted environmental samples is a poor indicator of its bioavailability, mobility and toxicity, as its environmental behaviour depends critically on its form. Metals can form various complexes with soil components, but only some of them are bioavailable. The behaviour of heavy metals in soil, and uptake by plants, is controlled by element speciation and by soil properties, such as pH, particle size, cation-exchange capacity, content of organic matter, content and type of clay minerals and Al, Fe and Mn oxides, and redox potential (Fijałkowski et al. 2012; Łukowski et al. 2013).

The sequential chemical extraction allows for operational fractionation of metals in solid samples, thus differentiates metal forms bound to different soil fractions. In the classic work of Tessier et al. (1979), the five-stage procedure was proposed to fractionate metals in river sediments. A harmonized, three-stage sediment sequential extraction protocol was established by the Community Bureau of Reference (BCR) of the Commission of the European Communities in 1993 (Quevauviller et al. 1994), while a modified BCR procedure was developed in 1999 (Rauret et al. 1999). Even this protocol is widely accepted and often used for fractionation of metals in various matrices, it still has some limitations (e.g. lack of specificity or species redistribution) as was discussed elsewhere (Bacon and Davidson 2008; Gleyses et al. 2002; Pérez et al. 2008). Moreover, this standardized protocol is very time consuming. In order to eliminate these shortcomings, sample treatment with microwaves (Castillo et al. 2011; Arain et al. 2008; Canepari et al. 2005; Garcia-Casillas et al. 2014; Reid et al. 2011; Ipolyi et al. 2002; Relić et al. 2013) or ultrasounds (Arain et al. 2008; Canepari et al. 2005; Garcia-Casillas et al. 2014; Krasnodębska-Ostręga et al. 2006; Rusnak et al. 2010; Relić et al. 2013) was proposed for single-step or, less frequently, for sequential extraction of metals from soil, sediment or sewage sludge samples.

The most beneficial effect of ultrasounds is the particle fragmentation and the micro-cracks that facilitate and accelerate many physicochemical processes, such as dissolution, digestion and extraction or leaching (Bendicho et al. 2012; Kazi et al. 2009). One should consider that during a sonication process, some properties of a sample may be changed and different fractionation patterns may be obtained in comparison to conventional shaking. However, such accelerated extraction process has been already used for fast monitoring the mobility, bioavailability and the eventual impact of anthropogenic heavy metals in environmental solid samples (Vaisanen et al. 2002). Different sources of ultrasounds, such as ultrasonic baths (Davidson and Delevoye 2001; Kazi et al. 2006; Krasnodębska-Ostręga et al. 2006) and probes (Davidson and Delevoye 2001; Greenway and Song 2002; Pérez-Cid et al. 1998), have been used for metal fractionation. Generally, the probe system, carried out by direct insertion of an ultrasonic probe into a suspension of the powdered material, provides more efficient extraction of analytes in shorter time (Davidson and Delevoye 2001). However, samples are treated by the ultrasound probe one by one, while in the ultrasound bath or microwave oven, many samples can be treated simultaneously.

The literature review reveals that the effect of ultrasound-assisted extraction was different for different solid samples and each element, and the recoveries of metals were often non-quantitative when compared to classical method or certified values of reference materials (Davidson and Delevoye 2001; Greenway and Song 2002; Pérez-Cid et al. 1999; Rusnak et al. 2010). Only a few papers have demonstrated achievement of metal amounts extracted by ultrasounds equivalent to those obtained by conventional standardized protocol, such as sequential extraction scheme. So far, the best results using ultrasonic bath (Kazi et al. 2006) or probe (Pérez-Cid et al. 1998) were obtained for sewage sludge. Kazi et al. (2006) has observed that, except of copper, the recoveries of Cd, Cr, Ni, Pb and Zn in steps 1–3 were in the range 95–117 %. The recoveries in the range 96–100 % were obtained for Cu, Cr, Ni, Pb and Zn in all fractions by Pérez-Cid et al. (1998), but it must be mentioned that the content of metals in some fractions (Cr and Pb in fractions I and II and Ni in fraction II) was not detectable by flame atomic absorption spectrometry (FAAS). The main sources of errors influencing analytical results were not identified so far.

The aim of this work was to develop a universal ultrasound-assisted sequential extraction (UASE) procedure for fractionation of several trace metals in soil. Therefore, natural soil samples of different physicochemical properties have been used within optimization of working parameters of the ultrasound probe. During optimization of the procedure, the modified BCR conventional sequential extraction (CSE) protocol (with the aid of a vertical rotor) was used for comparison. The developed UASE method of fractionation of Cd, Pb and Ni in soil with electrothermal atomic absorption spectrometric (ETAAS) detection was fully validated according to the international guidelines ISO/IEC 17025 (2005) and uncertainty budget was estimated. The certified reference material of lake sediment BCR-701 was used for trueness control. The method was applied for the determination of heavy metal fractions in soil collected from an arable layer in the province of Podlasie (Poland).

## Materials and methods

### Reagents and materials

Acetic acid, hydroxylammonium chloride, ammonium acetate and hydrogen peroxide (30 %) (pure for analysis) were obtained from POCh (Poland). Nitric acid and hydrochloric acid (Suprapur) were obtained from Merck (Germany). Standard solutions were prepared by gravimetric dilution of stock solutions of cadmium, lead and nickel (1000 μg mL^−1^, Fluka, Germany). Magnesium nitrate(V), palladium and ammonium dihydrogen phosphate, used as matrix modifiers, were obtained from Fluka (Germany). Ultrapure water was obtained from Milli-Q system (Millipore, USA).

Soil samples, various in terms of physicochemical properties (agricultural type, pH, content of organic matrix), were collected from the arable layer in the province of Podlasie (Poland). All soil samples, air-dried, were homogenized and sieved using a 1-mm sieve. Samples of mineral soil, light soil—L, medium soil—M and heavy soil—H, were used for optimization of the UASE procedure. Seven samples of light (L) and medium (M) mineral soil and 3 samples of organic (O) soil, used as arable land, were analysed using developed procedure. The physicochemical characteristics of collected soils are presented in Table 1.

**Table 1:** Characteristic of soil samples collected in the Podlasie Province (agricultural type of soil: L—light, M—medium, H—heavy, O—organic)

Sample	Type of soil	pH_KCl_	C_Corg_, %	Pseudo-total content of metal ± SD, mg kg^−1^		
				Cd	Pb	Ni
L	Brown	4.3	2.1	0.20 ± 0.01	7.3 ± 0.3	3.1 ± 0.1
M	Brown	7.1	2.0	0.40 ± 0.01	6.9 ± 0.2	6.9 ± 0.2
H	Brown	4.3	4.3	0.40 ± 0.01	13.9 ± 0.3	10.2 ± 0.5
L1	Brown	4.3	1.5	1.60 ± 0.04	11.9 ± 0.4	5.6 ± 0.2
L2	Brown	4.6	1.3	0.20 ± 0.01	9.8 ± 0.4	3.0 ± 0.1
M1	Brown	4.7	1.5	2.20 ± 0.06	8.5 ± 0.3	7.6 ± 0.3
M2	Brown	4.8	1.6	1.60 ± 0.05	7.3 ± 0.3	9.6 ± 0.2
M3	Podzols	6.0	2.8	0.70 ± 0.02	9.3 ± 0.2	7.0 ± 0.2
M4	Black	7.2	4.1	7.90 ± 0.31	8.8 ± 0.3	8.7 ± 0.2
M5	Black	7.3	3.0	0.40 ± 0.01	7.8 ± 0.3	9.8 ± 0.3
O1	Peat	5.2	24.8	2.10 ± 0.04	18.8 ± 0.9	8.1 ± 0.2
O2	Mud-and-peat	5.6	36.4	1.60 ± 0.07	28.6 ± 1.1	12.1 ± 0.3
O3	Muck-and-mineral	6.2	27.3	1.60 ± 0.05	22.5 ± 0.5	8.0 ± 0.3

Certified reference material of lake sediment BCR-701 (IRMM, Belgium) was used for trueness control within validation of the UASE procedure.

### Instrumentation and methods

An ultrasound processor, VCX 130 model (Sonics and Materials, USA) (max power 130 W, max frequency 20 kHz) equipped with titanium probe, was used in a pulsed mode (on/off, 15 s/15 s). In order to keep the constant temperature during the sonication process (*T* = 25 ± 5 °C), the system was cooled down with flowing tap water as described previously (Leśniewska et al. 2016).

Electrothermal atomic absorption spectrometer (Solaar M6, Thermo Electron Corporation) equipped with a Zeeman-effect background correction and graphite tubes with integrated Lvov’s platform were used for the determination of metal content. Hollow cathode lamps were operated as follows: for Cd (Thermo Scientific, UK) at 5 mA, for Pb (Photron, Australia) at 4 mA and for Ni (Narva, Germany) at 15 mA. The absorbance of metals in soil fractions was measured at 228.8 nm for Cd and at 217.0 nm for Pb with a spectral bandpass of 0.5 nm, while at 232.0 nm for Ni with a spectral bandpass of 0.2 nm. A palladium modifier (10 μL of 0.5 mg mL^−1^) was used for the determination of Cd in fraction I and fraction III, and a phosphate modifier (10 μL of 0.1 mol L^−1^ NH_4_H_2_PO_4_) was used for the determination of Cd in fraction II. Magnesium nitrate (10 μL of 0.5 mg mL^−1^) was used as a chemical modifier for Pb determination in all fractions. Due to very corrosive properties of hydroxylamine chloride towards graphite tubes, the extracts of fraction II were evaporated and the residues were dissolved in 0.1 mol L^−1^ nitric acid.

The following optimized heating programs were used for the determination of metals in soil fractions FI/FII/FIII: Cd—drying at 110 °C for 25 s, ashing at 1000/1200/1000 °C for 5 s and atomization at 1500/1800/1500 °C for 3 s; Pb—drying at 110 °C for 25 s, ashing at 800/900/900 °C for 20 s and atomization at 1600/2300/1900 °C for 3 s; and Ni—drying at 110 °C for 25 s, ashing at 1100/1300/1300 °C for 10 s, and atomization at 2450/2700/2600 °C for 3 s.

The pH of soil was measured in 1 mol L^−1^ KCl by a potentiometric method. In order to classify collected soil samples into proper agricultural type, the content of organic carbon was determined by the modified Tiurin’s method (Bednarek et al. 2004). The *aqua regia* procedure ISO 11466 (1995) was used for the determination of pseudo-total content of metals in soil.

### Ultrasound-assisted sequential extraction procedure

The modified BCR procedure, conventional and ultrasound-assisted, was used for fractionation of metals in soil. In brief, for ultrasonic extraction of water-, acid-soluble, and exchangeable metal fraction (FI), 40 mL of 0.11 mol L^−1^ CH_3_COOH was added to 1 g of soil sample and sonicated with the ultrasound probe for 7 min at 15 W. For extraction of reducible metal fraction (bound to iron and manganese hydroxides) (FII), 40 mL of 0.5 mol L^−1^ NH_2_OH·HCl (pH 1.5) solution was added to the soil residue and sonicated for 10 min at 15 W. The organically bound metal fraction (FIII) was released by oxidation of the organic matter using 10 mL of 30 % H_2_O_2_ (pH 2) and sonication of the suspension for 4 min at 15 W, next heating for 1 h at 85 °C and re-extraction of mineralization products with 50 mL of 1 mol L^−1^ CH_3_COONH_4_ (pH 2) and sonication for 6 min at 15 W. The suspension was always centrifuged at 3000 rpm for 15 min. Before the next extraction step, the remaining solid residue was washed with 20 mL of ultrapure water.

## Results and discussion

### Optimization of the UASE procedure

The extraction efficiency of metals from solid samples depends on the type of ultrasound processor, its power or frequency and sonication time, as well as the type of sample. In order to develop the UASE procedure useful for simultaneous fractionation of Cd, Pb and Ni in soil, the working conditions of ultrasound probe were optimized individually for each step of procedure. The extraction was performed at least in triplicate. Moreover, different types of mineral soil (light—L, medium—M and heavy—H) were used during the optimization process. The results obtained by accelerated procedure were always compared with the results obtained in the same soil by CSE modified BCR procedure using reagents recommended in the original protocol, and the recoveries of metals were calculated. During the selection of optimal sonication conditions, the parameters chosen previously for fractionation of Cu (Leśniewska et al. 2014) in soil were also taken into consideration.

**Fig. 1:**
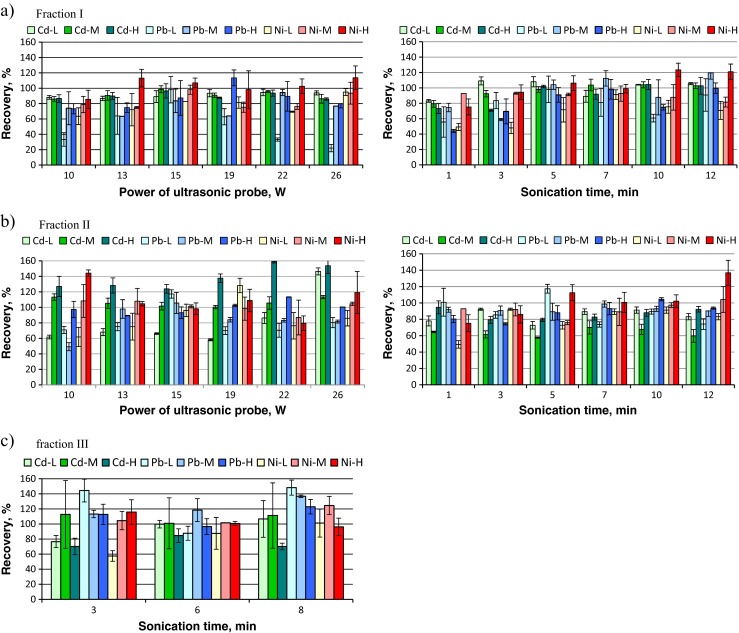
Recovery of Cd, Pb and Ni in fractions extracted from soil in dependence of the power of ultrasonic probe (fixed sonication time 5 min) and the sonication time (fixed sonication power 15 W): **a** fraction I, **b** fraction II, **c** fraction III; (*L*—light soil, *M*—medium soil, *H*—heavy soil)

The influence of a power of ultrasound probe on the recovery of metals in fraction I and fraction II was studied in the range from 10 to 26 W (amplitude from 50 to 100 %) at sonication time of 5 min (Fig. 1). In fraction I, the recoveries of metals from all studied samples were in the range 63–114 %, except of the low recovery of Pb from light soil (22–98 %). The recoveries approaching 100 % for all metals (mean recovery equal to 93.5 ± 7.7 %) were obtained using the power of ultrasound probe of 15 W. The same power was used previously for fractionation of Cu in soil (Leśniewska et al. 2014). Hence, in the next step, the sonication time was altered in the range 1–12 min using ultrasound power of 15 W. As can be seen in Fig. 1a, the sonication for 1 or 3 min was too short to extract all metals from soil samples. The effective recovery of Cd was obtained using 5 min sonication time (with mean value for all soil samples of 102.5 %), while for Pb and Ni, more efficient recoveries were obtained using 7 min sonication (97 % for Pb and 94 % for Ni). Longer ultrasonic treatment of samples, 10 min for Pb (L, M and H soil) and 10 and 12 min for Ni (L and M soil), resulted in partial re-adsorption of Pb and Ni on soil particles, what was also demonstrated by Pérez et al. (2008). For the next study, the treatment of soil with ultrasounds for 7 min at power of 15 W was chosen for simultaneous extraction of Cd, Pb and Ni into an acid-soluble fraction (FI). The recoveries of metals released from soil samples under optimized working conditions are higher than those obtained by Canepari et al. (2005) and comparable to results obtained by Kazi et al. (2006) and Pérez-Cid et al. (1998).

The highest recoveries of Cd, Pb and Ni in fraction II were obtained using the ultrasound probe working at 15 W (Fig. 1b). Under these conditions, the recoveries of Ni and Pb were in the range of 93–117 % (with mean recovery value of 102 ± 9 %). More complex situation was observed for extraction of Cd; as for sample L, the recovery was low, in the range 60–80 %, while for sample H, the recovery was very high, in the range 120–160 %. Such high results obtained for sample H were probably an effect of re-adsorption of Cd in the first step of extraction (Penilla et al. 2005; Pérez et al. 2008). The reason for low recovery of Cd in sample L is unknown, especially in view of the fact that the affinity of Cd to ≡FeOH is low (log *β* = −2.22). Optimization of sonication time has shown that the highest recoveries of all metals were obtained using 10 min sonication (Fig. 1b). Therefore, for extraction of metals bound to reducible fraction (FII), the sonication of samples for 10 min at 15 W was chosen. Such conditions provided quantitative recovery and good repeatability of the results for Pb, Ni and Cu and Zn. The recovery of Cd was lower and dependent on the type of soil sample.

On the basis of our previous results (Leśniewska et al. 2014), only the second step of releasing of metals from oxidazible fraction, namely the sonication time required for efficient re-extraction of mineralization products with CH_3_COONH_4_, was optimized in this work. The power of ultrasound probe (15 W) applied in this step was the same as used before. The influence of sonication time on the recovery of metals is presented in Fig. 1c. The best recovery of all metals in FIII was achieved using 6 min sonication.

The important advantage of UASE procedure is short extraction time necessary for fractionation of Cd, Pb and Ni in soil, which is only 87 min (including 27 min of sonication), especially when compared to 51 h (48 h of extraction) of the original time of extraction according to CSE modified BCR procedure. The additional benefit of the developed method is that the same protocol is also suitable for fractionation of Cu.

The comparison of results obtained for Cd, Pb and Ni by conventional and UASE procedures in various soil samples (L, M and H), expressed as recovery, is outlined in Table 2. Even some differences between light, medium and heavy soils have been observed; the recoveries of all metals were in the range of 81–113 %. In order to confirm the accuracy of results obtained by means of the above procedures, they were applied to the analysis of certified reference material BCR-701 (lake sediment). As can be seen in Table 3, the results obtained for all metals by conventional procedure are generally in good agreement with certified values (recoveries in the range 92–113 %), while those obtained by accelerated procedure are slightly lower (especially for Ni in FI) (recoveries in the range 82–105 %). These discrepancies are probably an effect of different particle sizes and type of analysed material (sediment vs. soil) during procedure optimization. Nevertheless, concentrations of all metal fractions in BCR-701 determined by UASE procedure are within the results reported in the literature and compiled by Sutherland (2010). Good agreement of the results with certified values was also obtained for overall metal recoveries in fractions FI-FIII (93–103 %), except for Ni extraction (89 %), that was also reported by Pérez et al. (2008) and Ipolyi et al. (2002). These studies indicate a great potential of UASE procedure for fast monitoring of mobile metal fractions in soil. However, the re-adsorption phenomenon suggests that metal distribution has to be cautiously interpreted, principally that of Pb and Ni. Even though the above described procedure provided accurate results, it requires validation before any further application.

**Table 2:** Recovery of Cd, Pb and Ni in fraction of soil extracted according to the developed ultrasound-assisted method in comparison to the conventional modified BCR method

Soil sample	Fraction	Recovery ± SD, % (*n* = 3)		
		Cd	Pb	Ni
L—light soil (pH 4.3)	F I	89.1 ± 7.7	81.2 ± 12.9	91.3 ± 6.3
	F II	91.3 ± 3.9	89.5 ± 3.1	91.3 ± 3.9
	F III	99.8 ± 5.0	87.7 ± 9.3	86.6 ± 3.0
M—medium soil (pH 7.1)	F I	103.8 ± 7.5	112.4 ± 9.9	92.6 ± 9.9
	F II	95.2 ± 3.4	92.8 ± 3.2	97.4 ± 2.9
	F III	100.9 ± 15.8	113.4 ± 15.2	101.5 ± 6.8
H—heavy soil (pH 4.3)	F I	87.6 ± 8.8	98.3 ± 2.7	99.0 ± 5.5
	F II	88.0 ± 4.2	104.6 ± 2.0	102.2 ± 7.7
	F III	84.6 ± 9.0	96.6 ± 10.4	100.6 ± 2.7

**Table 3:** Comparison of the results for CRM BCR 701 by the conventional sequential extraction (CSE) method and ultrasound-assisted sequential extraction (UASE) method

	Content ± U, mg kg^−1^ (*k* = 2)		Recovery, %	Content ± U, mg kg^−1^ (*k* = 2)	Recovery, %
	Certified value	CSE method		USAE method	
Fraction I					
Cd	7.34 ± 0.35	7.40 ± 1.70	100.8	6.81 ± 0.83	92.8
Pb	3.18 ± 0.21	3.61 ± 0.36	113.5	3.3 ± 0.36	103.8
Ni	15.4 ± 0.9	15.1 ± 1.6	98.0	12.6 ± 2.1	81.8
Cu	49.3 ± 1.7	53.0 ± 5.2	107.5	40.2 ± 5.0	81.6
Fraction II					
Cd	3.77 ± 0.28	4.10 ± 0.82	108.7	3.75 ± 0.42	99.5
Pb	126 ± 3	128 ± 13	101.6	129 ± 8	102.4
Ni	26.6 ± 1.3	27.6 ± 3.4	103.7	24.2 ± 2.8	91.0
Cu	124 ± 3	132.5 ± 10.2	106.8	117.2 ± 8.6	94.5
Fraction III					
Cd	0.27 ± 0.06	0.30 ± 0.08	111.1	0.29 ± 0.07	107.4
Pb	9.3 ± 2.0	10.2 ± 2.5	109.7	9.8 ± 2.3	105.4
Ni	15.3 ± 0.9	14.1 ± 2.0	92.2	14.1 ± 1.7	92.2
Cu	55.2 ± 4.0	61.5 ± 8.8	111.4	56.1 ± 7.8	101.6
Sum of fractions I+II+III					
Cd	11.38	11.81	103.7	10.85	95.3
Pb	138.48	142.21	102.7	142.1	102.6
Ni	57.3	57.1	99.7	50.9	88.8
Cu	228.5	247	108.1	213.5	93.4

### Validation of the UASE procedure

The validation of the developed UASE procedure for fractionation of Cd, Pb and Ni in soil was performed according to the international guidelines ISO/IEC 17025 (2005). Various analytical parameters, such as linearity, limit of detection (LOD) and limit of quantification (LOQ), precision, selectivity and trueness, were estimated.

For the evaluation of the linearity of calibration graphs, the standards of Cd, Pb and Ni in the extraction solutions were prepared. The linearity of the calibration graph was considered acceptable when the correlation factor was higher than 0.995. The sensitivity of measurements of Cd and Pb (expressed as a slope of calibration graph) was dependent on the type of extraction solution, being the highest for fraction II (in diluted HNO_3_ after evaporation of NH_2_OH HCl). This phenomenon affected the linear range of calibration graphs of both metals. Such effect was not observed for measurements of Ni (Table 4). The selectivity of the method was evaluated by the comparison of calibration graphs obtained by external calibration procedure (reagent-matched standard solutions) and standard addition method (extract of soil spiked with increasing amounts of analyte). As the slopes of calibration graphs obtained by these techniques were the same in the range of analytical error, the external calibration procedure was used for quantification of metal fractions in soil by the ETAAS technique. The LOD was calculated according to the following equation: LOD = blank + 3SD_blank_, where the extraction solution was used as the blank sample. The LOQ was calculated as LOQ = blank + 6SD_blank_. In order to assess these parameters for soil samples, the volume of extraction solution and the mass of soil sample were used for calculations.

**Table 4:** Validation parameters for the ultrasound-assisted extraction method for determination of Cd, Pb and Ni fractions in soil by the ETAAS technique

Validation parameter	Soil fraction: extraction solution								
	F I: 0.11 mol L^−1^ CH_3_COOH			F II: 0.5 mol L^−1^ NH_2_OH HCl^a^			F III: 1 mol L^−1^ CH_3_COONH_4_		
	Cd	Pb	Ni	Cd	Pb	Ni	Cd	Pb	Ni
Linear range of calibration graph, ng mL^−1^	0.3–10	0.85–25	3–120	0.1–4	1.3–20	4–100	0.1–10	2.3–20	5–120
Slope of calibration graph	0.0333	0.0135	0.0031	0.0911	0.0164	0.0033	0.0475	0.0147	0.0032
Regression coefficient, *r*	0.9997	0.9996	0.9996	0.9994	0.9996	0.9996	0.9975	0.9982	0.9992
Limit of detection for extraction solution, ng mL^−1^	0.06	0.52	2.0	0.06	0.62	3.1	0.02	1.53	3.5
Limit of quantification for extraction solution, ng mL^−1^	0.29	0.85	2.8	0.12	1.31	3.9	0.06	2.31	5.1
Precision of absorbance measurements for soil extract as RSD, % (*n* = 6)	2.2	2.0	2.6	2.8	3.3	2.9	2.2	3.7	2.5
Limit of detection for soil fraction, ng g^−1^	2.5	20	81	2.4	25	122	1.0	76	175
Limit of quantification for soil fraction, ng g^−1^	11.5	34	140	4.9	52	195	3.0	92	255
Repeatability of metal determination in BCR 701 fraction as RSD, %, (*n* = 6)	4.4	5.6	12.5	5.3	2.2	6.6	3.4	3.8	2.8
Trueness of the procedure^b^ Found content ± *U*, *k* = 2, mg kg^−1^	6.81 ± 0.83	3.30 ± 0.36	12.6 ± 2.1	3.75 ± 0.42	128.6 ± 8.2	24.2 ± 2.8	0.290 ± 0.068	9.8 ± 2.3	14.1 ± 1.7
Bias, %	−7.2	3.8	−18.2	−5.3	2.1	−9.0	7.4	−3.2	−7.8

^a^After evaporation and dilution in 0.1 mol L^−1^ HNO_3_

^b^As compared to certified value of BCR 701

The precision of measurements of analyte absorbance in extraction solutions, defined as a degree of agreement between a set of results, was assessed by six independent measurements of the same sample. It was expressed as the relative standard deviation (RSD) and gave values below 2.9 %. The repeatability of extraction of metals from soil samples and BCR-701 by the UASE method was evaluated on the basis of six independent extractions of the same sample under the same condition in a short period of time. It was expressed as RSD and gave values in the range 2–13 % for soil and 2–7 % for BCR-701. Better repeatability obtained for certified reference material (CRM) results from better homogeneity of this material. The limits of detection and quantification of metals in soil fractions are even 40–50 times higher than those obtained for pure extraction solutions.

The trueness of the developed procedure of sequential extraction of Cd, Pb and Ni, defined as a closeness of the mean value of obtained results to the true value, was evaluated by analysis of BCR-701. The content of Cd, Pb and Ni in all fractions was compared with the certified values and the bias of the UASE method was evaluated. The bias of the method was calculated as a difference between the mean value of the obtained results and the reference value. The highest bias of the method was obtained for fractionation of Ni (from −18.2 % in FI to −7.8 % in FIII), but for other metals, was below 7.5 %. All results are within the range of data for BCR-701 compiled from 33 literature data sets (Sutherland 2010). The validation parameters of the developed UASE method are summarized in Table 3.

Evaluation of expanded uncertainty of Cd, Pb and Ni content in soil fractions by the developed UASE procedure was performed in accordance with the Guide to the Expression of Uncertainty in Measurement (2008) using a modelling approach, similarly to the scheme presented in Leśniewska et al. (2016). For that purpose, possible sources of uncertainty of the measurement procedure were identified and individual standard uncertainties of these components were estimated. The combined standard uncertainty of results was calculated according to the law of an uncertainty propagation; next, the uncertainty budget was estimated. For calculation of the content of metals in soil fractions, the following model equation was used:

$$ {c}_{\mathrm{Me}}=\frac{\left(\frac{A_{\mathrm{s}}-a}{b}\right)\cdot {V}_{\mathrm{e}}\cdot f}{m_{\mathrm{s}}\cdot R} $$

where *c*_Me_ denotes the metal content in fraction of soil (mg kg^−1^), *A*_s_ is the absorbance of analyte in given soil extract, *a* is the intercept of the calibration graph, *b* is the slope of the calibration graph, *V*_e_ is the volume of given soil extract (L), *f* is the dilution factor of extract, *m*_s_ is the mass of soil (g) and *R* is the recovery of analyte in fraction of BCR-701.

The parameters included in the model equation were identified as components significantly contributing to the measurement result. On the basis of their individual standard uncertainties and the law of propagation of uncertainty, the combined standard uncertainty of analyte content in fractions of soil *u*_c_(*c*_Me_) was evaluated according to the following equation:

$$ \frac{u_{\mathrm{c}}\left({c}_{\mathrm{Me}}\right)}{c_{\mathrm{Me}}}=\sqrt{{\left(\frac{u\left({m}_{\mathrm{s}}\right)}{m_{\mathrm{s}}}\right)}^2+{\left(\frac{u\left({V}_{\mathrm{e}}\right)}{V_{\mathrm{e}}}\right)}^2+{\left(u\left(\mathrm{cal}\right)\right)}^2+{\left(\frac{u(R)}{R}\right)}^2+{\left(\frac{u(f)}{f}\right)}^2+u{\left(\mathrm{repeat}{.}_{\mathrm{e}\mathrm{xtr}.}\right)}^2} $$

where *u(m*_s_ *), u(V*_e_ *)*, *u*(cal), *u*(*R*), *u*(*f*), and *u*(repeat._extr*.*_) denote standard uncertainties of mass of soil, volume of extract, calibration, recovery, dilution factor and repeatability of extraction process, respectively.

To obtain an expanded uncertainty (*U*) of the result at the 95 % confidence level, the combined standard uncertainty of analyte content in fractions was multiplied by the coverage factor *k* of 2. The expanded uncertainties of content of metals in fractions FI, FII and FIII obtained for the same samples using the modified BCR method with conventional sequential extraction (CSE) were calculated accordingly.

**Fig. 2:**
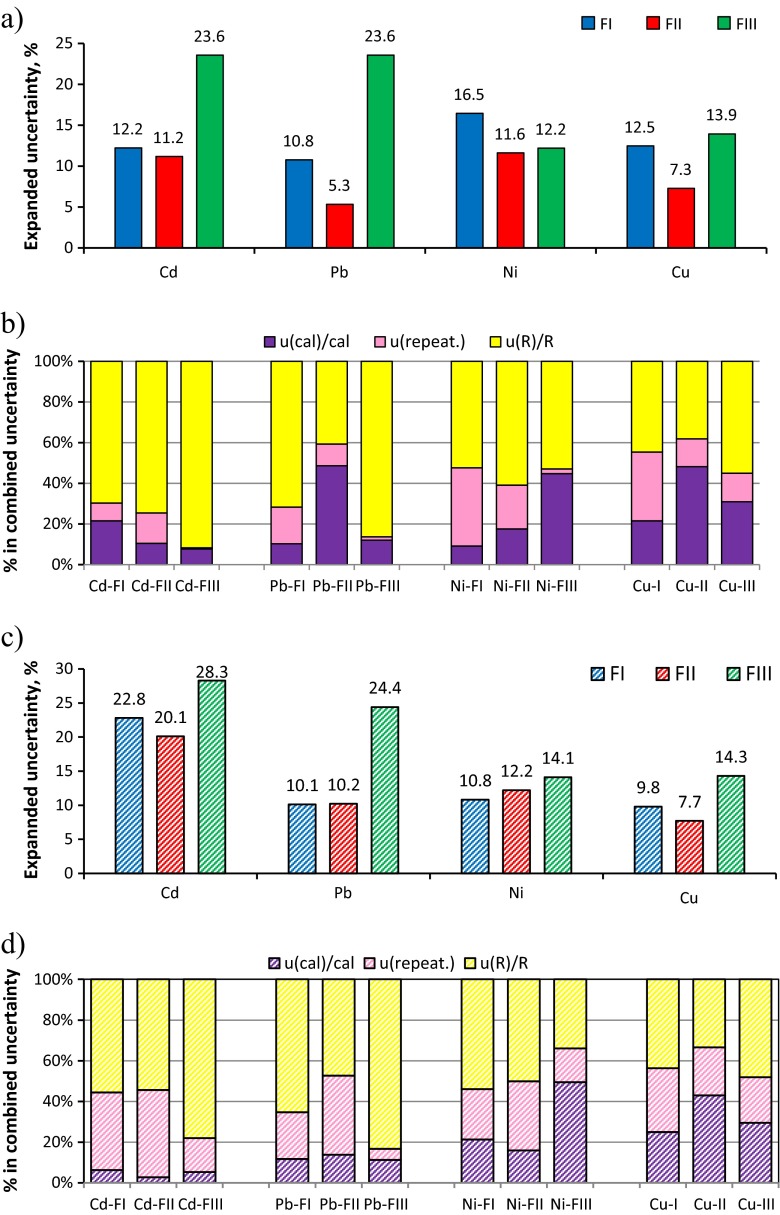
Uncertainty budget: Expanded uncertainty (in %, *k* = 2) of content of metals in fraction I, II and III determined (**a**) after ultrasound-assisted extraction of soil and (**c**) after conventional modified BCR procedure. The percentage contribution of uncertainty of component in a combined uncertainty of content of metals in fraction of soil determined (**b**) after ultrasound-assisted extraction of soil and (**d**) after the conventional modified BCR procedure; (*u*(*m*_s_)/*m*_s_, *u*(*V*_e_)/*V*_e_, *u*(cal)/cal, *u(R)/R, u*(*f*)/*f*, and *u*(repeat._extr._) denote relative uncertainty of mass of soil, volume of extract, calibration, recovery, dilution factor and repeatability of extraction process; *u*(*m*_s_)/*m*_s_ and *u(V*_e_)/*V*_e_ were <0.1 %; *u*(*f*)/*f* < 0.5 %)

The values of relative uncertainty of each component in combined uncertainty and expanded uncertainty of content of metals in fractions FI, FII and FIII are presented in Fig. 2 and Table 3. In order to study capability of the developed UASE procedure, the uncertainties of results of fractionation of Cu in soil obtained in our earlier study (Leśniewska et al. 2014) have been also calculated (Fig. 2 and Table 3). The highest expanded uncertainty of results was observed for content of metals in FIII (12–24 %), while for FI and FII, was in the range 5–16 % (Fig. 2a) that is similar to the pattern represented for BCR-701 and resulted from the complexity of the sequential extraction procedure. A distribution of expanded uncertainty of content of metals in soil fractions obtained by the CSE method was analogous. Again, the highest values of expanded uncertainty were obtained for metal content in fraction III (14.1–28.3 %). However, for CSE procedure, the expanded uncertainty of Pb, Ni and Cu content in FI was lower, probably due to higher recoveries of these metals in FI.

Finally, the uncertainty budget was constructed in order to indicate critical control points of the developed UASE and CSE methods. The percentage contribution of uncertainty of each component in combined uncertainty was calculated (as, e.g. [*u*(*m*_s_)/*m*_s_]^2^/[*u*_c_(*c*_Me_)*/c*_Me_]^2^). As can be seen in Fig. 2b, d, the uncertainty of metal content in all fractions is strongly influenced by their recoveries (38–92 % for UASE and 33–83 % for CSE), calibration (9–48 % for UASE and 3–49 % for CSE) and repeatability of extraction step (1–38 % for UASE and 5–43 % for CSE). Uncertainties of the mass of sample, the volume of extract and the dilution factor were neglected in combined uncertainty of metal content in all cases as their values were below 0.5 %. The largest shares in uncertainties of recovery of analytes derive from the uncertainties of certified values of CRM (Table 3). However, low recoveries of Cd, Ni and Cu in FI obtained by using the UASE method influenced uncertainties of their content. The second important source of uncertainty was the concentration of analytes in the extract of soil that was calculated from the calibration graph. Among the three sources of uncertainty in the calibration step, a slope of the calibration graph and repeatability of measurements of analyte absorbance had significant effect on the determined concentration of analytes. Preparation of standard solutions for calibration by dilution of stock standard solutions introduced the lowest uncertainty in that step. Repeatability of the extraction step significantly affected the combined uncertainty of metal content in all fractions of soil (Fig. 2b, d). Such effect was strongly observed when the CSE procedure that was applied as contribution of *u*(repeat.) in the combined uncertainty was in the range 16–43 % almost for metals in all fractions, whereas for developed UASE, its share was mostly below 15 %.

### Application of the UASE method

**Fig. 3:**
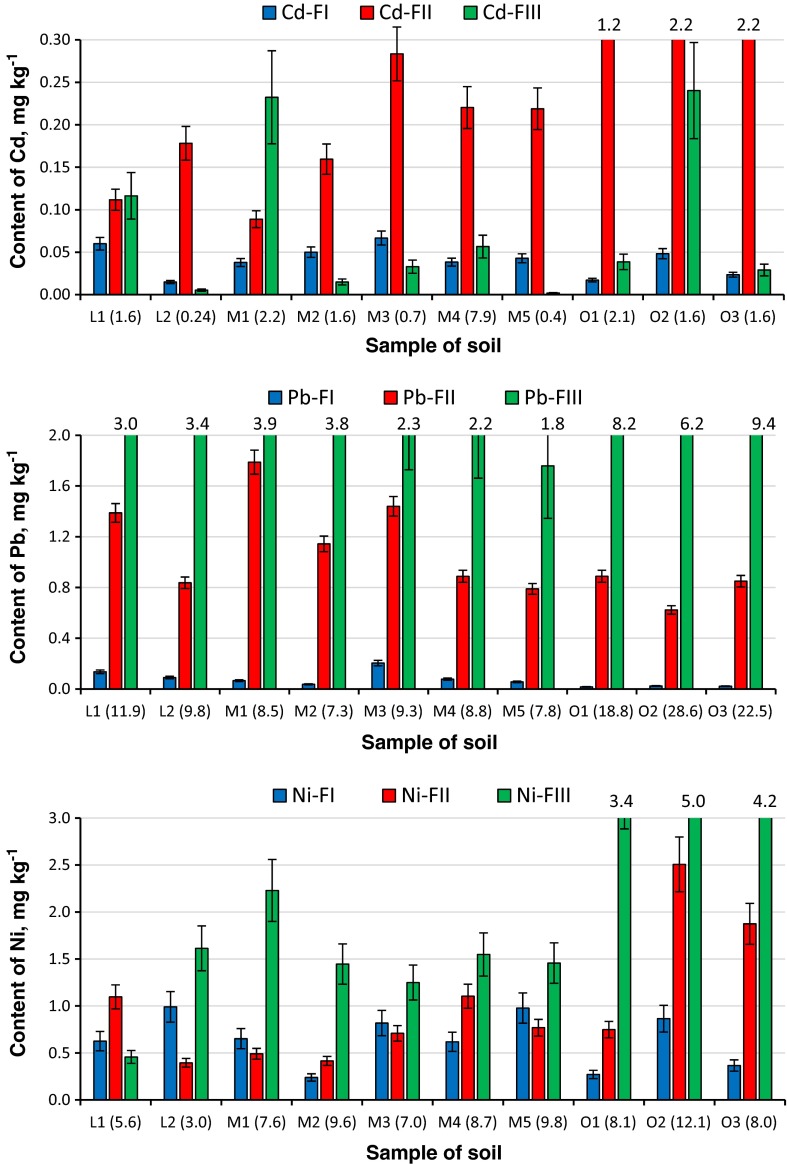
Content of Cd, Pb and Ni in fractions of soil collected from the Podlasie Province after ultrasound-assisted extraction with expanded uncertainty (*k* = 2) (soil category: *L*—light, *M*—medium, *O*—organic; in *brackets*, the pseudo-total content of metal in mg kg^−1^)

The developed fast UASE method was applied to fractionation of Cd, Pb and Ni in ten samples of soil collected from arable land of the province of Podlasie. The content of analyte fractions in soils as well as the pseudo-total content of metals in soils after *aqua regia* digestion was determined by ETAAS (Fig. 3 and Table 1). The pseudo-total content of metals determined in all analysed samples did not exceed its permissible limit for agricultural soil in Poland, which was set at 4 mg kg^−1^ for Cd, 100 mg kg^−1^ for Pb and 100 mg kg^−1^ for Ni (Ordinance of the Minister of Environment, Poland 2002). Therefore, the analysed soil samples were considered as unpolluted. The content of metals in organic soils was generally higher than in mineral soils. Distribution of metals among soil fraction was different for various elements, what is consistent with literature data. The highest content of Cd was determined in the reducible fraction (FII) and lower in fraction III and fraction I. In case of Pb, the highest content was observed in fraction III, while the lowest in fraction I. The Ni content in fractions of mineral soil is similar, while for organic soil, its highest content was determined in FIII. The distribution of metals among fractions in soils of the Podlasie Province is not discussed in detail as these issues are beyond the scope of this work and due to variety of properties of analysed soil (Table 1). However, it is worth to point out that the results of fractionation of four metals in soil can be achieved within 2 h (taking into account 87 min for extraction of metals from soil and 30 min for their determination by AAS), indicating usability of the procedure for fast screening of mobile metal fractions.

## Conclusions

In order to facilitate the method for determination of Cd, Pb and Ni fractions in soil, a fast ultrasound-assisted sequential extraction (UASE) procedure based on modified BCR protocol was proposed. Application of ultrasounds allowed to shorten the total time of procedure from 51 h to 87 min (27 min of sonication of soil and 1 h heating at 85 °C).

During method validation, it was found that external calibration technique using sample-matched solutions is appropriate for quantification of elements in soil extracts. The trueness of the developed method assessed by analysis of BCR-701 was acceptable, varying for different metals and fractions. The extended uncertainty of content of metal fractions in soil was in the range of 5.3–23.6 %, being the highest for fraction III. The values of extended uncertainty obtained for the developed UASE procedure were generally lower than for the CSE modified BCR procedure. The analysis of uncertainty budget indicated that the main share in uncertainty of results derived from the evaluation of the recovery of metals in CRM and the quantification of metals in soil extracts (including the calibration step). The recovery values were affected by the analyte content, as well as the type and homogeneity of analysed samples. The significant share in combined uncertainty of results had the standard uncertainty of certified value of metals in CRM. Unfortunately, a CRM for sequential extraction with lower uncertainty of reference values is unavailable.

In our work, ultrasonic treatment caused lower amounts of Pb and Ni to be released in the first fraction of soil. These results are probably an effect of re-adsorption of metals on the surface of soil during the extraction step, which is enhanced when ultrasounds are applied. Apart of a better penetration of solvent into the solid sample to extract the metal, the ultrasounds cause the activation of adsorptive sites of soil particles. However, the results obtained for fractionation of elements in BCR-701 were in the range of results published by others (Sutherland 2010). The metal distribution in soil has to be cautiously interpreted, but the procedure is very suitable for fast screening of mobile metal fractions in soil, so it can be recommended for eco-toxicological studies and environmental risk assessment.
